# Clinical Cases from My Note-Book

**Published:** 1868-02

**Authors:** N. S. Davis

**Affiliations:** Professor of Practical and Clinical Medicine, in Chicago Medical College


### CLINICAL CASES FROM MY NOTE-BOOK.

*Neuralgia of the Rectum.—False Pains in the Uterus.—Effects of Belladonna.*

By N. S. Davis, M.D., Professor of Practical and Clinical Medicine, in Chicago Medical College.

Case I. Mr. M., a laborer, residing on Sebor Street, was attacked rather suddenly with severe pain in the rectum, while at his work, June 10th, 1863. I was called to see him on the following day, when I found him in bed, with no fever, only slight quickening of the pulse, no coating on the tongue, and temperature natural, but suffering excruciating pain in the rectum. It was accompanied by no tenesmus or straining at stool, and no outward swelling. The interior of the rectum was very sensitive to the touch, but there were no hemorrhoidal tumors within reach of the finger, and no point of hardness and swelling, such as would indicate cellular inflammation tending to the formation of an abscess. Such acts as coughing, straining, or passing of wind from the rectum, rendered the pain more severe.

The patient was directed to remain at rest, and take eight grains of Dover's powder every three hours until the pain was relieved. The next day, I found him still suffering from the pain, unchanged, with the addition of nausea and moderate dysuria. I now directed warm narcotic fomentations to the perineum and hypogastrium, and, instead of the Dover's powder, gave sulphate of morphia, one-third of a grain, with bicarbonate of soda, five grains, every four hours. The following day, I found him still suffering paroxysms of extreme pain in the same locality as before, with extreme nausea and efforts at vomiting, cool extremities, and great sense of prostration. Being satisfied that most of the nausea and prostration was occasioned by the opiates, I omitted their further use and directed the following treatment:

℞. Nitrous Ether,-------------------------- ℨij.

Tinct. Belladonna,---------------------- ʒij.

Mix, and give a teaspoonful in sweetened water every 4 hours.

Also twenty drops of tincture of belladonna in half a teacupful of milk-warm water, to be used as an enema, and repeated every three or four hours until the pain was relieved, or the pupils became dilated from the effects of the belladonna. This treatment was followed by very prompt relief to both the nausea and the pain. Only two enemas were used, and after the belladonna and nitrous ether had been continued two days, no further treatment was required.

Case II. Mr. H., a young man, of good habits but nervous temperament, the night after having sat on the damp ground, was attacked with extreme paroxysmal pains in the rectum, with tenderness to pressure at the lower end of the coccyx, but no swelling or redness.

The intestinal evacuations had been previously regular, and there was present no decided febrile disturbance of the system. Any motion of the body by which the rectum was disturbed, such as coughing or the passage of wind from the bowels, greatly aggravated the pain. The local tenderness near the lower end of the coccyx, led me to regard the case, at first, as one of cellular inflammation, such as usually results in the formation of an abscess, afterwards followed by a fistula. I consequently enjoined rest, narcotic fomentations to the anus, and gave pulv. Doveri internally, to procure sleep. After pursuing this treatment for two days, without any decided change in the symptoms, I caused the bowels to be moved by a saline cathartic, the operation of which was accompanied by severe rectal pains, and followed by no relief to the patient. Enemas, containing tincture of opium, were used after the bowels had been evacuated, but afforded partial relief only so long as their effects were sufficient to stupefy the patient. After the patient had been under treatment three full days, finding the pain and morbid sensitiveness of the rectum nearly the same as at first, and yet no point of swelling and hardness in the vicinity of the anus, such as always indicates the approach of an abscess, I

became satisfied that the case was one of neuralgia, and directed the following treatment:

℞. Chloroform, ---------------------------- ʒiij.

Tinct. Belladonna, ----------------------- ʒiij.

Syrup of Orange Peel, ------------------- ℥iij.

Mix, and take a teaspoonful every two hours, until the pupils become slightly dilated, when the interval between the doses should be increased to four hours.

He was also directed to have 20 drops of the tincture of belladonna in half a teacupful of warm water injected into the rectum each morning and evening. Under this treatment, the pain almost entirely subsided during the first twenty-four hours, and in three days the patient was able to leave the house, quite well. In two other cases, of similar character, relief was speedily obtained by the internal use of an equal mixture of tinct. belladonna and tinct. gelsemium, taken in doses of 20 drops every two or three hours.

Case III. Mrs. H., aged 35 years, of a nervous temperament and somewhat scrofulous diathesis, six months advanced in pregnancy, was attacked with severe paroxysmal pains in the rectum, which were accompanied by a disposition to force down, as in the pains of labor. She had been subject to annoyance from hemorrhoids, at times, for several years, and a tendency to constipation. But neither of these existed at that time sufficiently to account for the pains then existing. The following day, the pain in the rectum increased, and the "bearing down" became so regularly paroxysmal and severe, that the patient was induced to think that premature labor was actually taking place, and I was called in.

On making a vaginal examination, the os uteri was found undilated and firm, but the forcing pains that came every five minutes plainly crowded the whole uterus down lower in the cavity of the pelvis and caused a perceptibly increased rigidity of its neck. After a few hours delay, finding no change in the condition of the uterus, and the pains in the rectum increasing to a degree of great intensity, I became satisfied that there were no true labor pains, but the whole amount of suffering was occasioned by neuralgia or, at least, nervous irritation of the pelvic viscera. Knowing, from previous attendance upon the patient, that the exhibition of any of the ordinary preparations of opium would be speedily followed by persistent nausea and distressing vomiting, even when taken into the rectum, I at once prescribed an equal mixture of tincture of belladonna and tincture of gelsemium, of which 20 minims was to be taken every two hours, and 20 minims in two ounces of slightly warm water to be used as an enema. In less than two hours the pains were greatly lessened, and in six hours both pains and tenderness or morbid sensibility over the lower part of the abdomen and region of the rectum had entirely disappeared. But the belladonna had caused some dryness of the fauces, and sufficient dilatation of the pupils to cause some confusion of vision. The doses were now reduced to 15 minims, and given at intervals of four hours.

The following day, finding the patient free from pain, but feeble and entirely without appetite, I directed for her 8 minims of hydrochloric acid, to be taken in a tablespoonful of sweetened water four times a-day, instead of the belladonna and gelsemium, but to repeat the enema of warm water and belladonna whenever the pelvic or rectal pains should return. The acid had a good effect on the appetite and digestion, and the enema was required but two or three times during the succeeding three days. In the meantime, the contents of the bowels were evacuated naturally, and the patient passed the remainder of her period of gestation with but little trouble, and was confined at the proper time.

Knowing the influence of belladonna in relaxing the os uteri when it remains rigid after labor has commenced at the full period of pregnancy, I hesitated in regard to the propriety of using it in the foregoing case at the sixth month, lest it should increase the danger of a premature labor. The result, however, showed that my fears were not well founded.

